# Impact of sex on the prognosis of patients with esophageal squamous cell cancer underwent definitive radiotherapy: a propensity score-matched analysis

**DOI:** 10.1186/s13014-019-1278-0

**Published:** 2019-05-02

**Authors:** He-San Luo, Hong-Yao Xu, Ze-Sen Du, Xu-Yuan Li, Sheng-Xi Wu, He-Cheng Huang, Lian-Xing Lin

**Affiliations:** 1 Department of Radiation Oncology, Shantou Central Hospital, Affiliated Shantou Hospital of Sun Yat-sen University, Shantou, Guangdong China; 2 Department of Surgical Oncology, Shantou Central Hospital, Affiliated Shantou Hospital of Sun Yat-sen University, Shantou, Guangdong China; 3 Department of Medical Oncology, Shantou Central Hospital, Affiliated Shantou Hospital of Sun Yat-sen University, Shantou, Guangdong China

**Keywords:** Sex, Esophageal squamous cell cancer, Definitive radiotherapy, Propensity score-matched

## Abstract

**Background:**

The impact of sex on prognosis of patients with esophageal squamous cell cancer (ESCC) who underwent definitive radiotherapy remained unclear. The present study aimed to determine the impact of sex on the prognosis of patients with ESCC underwent definitive radiotherapy.

**Methods:**

Between January 2009 and December 2015, patients with ESCC underwent definitive radiotherapy in Shantou Central Hospital were included in this study. The Progression-free survival (PFS) and overall survival (OS) rates were estimated using the Kaplan-Meier method. The PFS and OS were compared between female and male patients. The Cox regression model was used to identify prognostic factors. Propensity score-matched analysis was performed to balance baseline characteristics between female and male patients.

**Results:**

A total of 683 ESCC patients treated with definitive radiotherapy were included, with 497 male and 186 female patients. In the whole cohort, female patients had a significantly longer median PFS (14.0 months vs 10.6 months, *P* = 0.0001, HR = 0.688, 95% CI, 0.567–0.836) and OS (20.8 months vs 15.9 months, *P* = 0.0005, HR = 0.702, 95% CI, 0.575–0.857). In the matched cohort, female patients still had a significantly longer median PFS (13.5 months vs 11.6 months) and OS (19.6 months vs 16.1 months). Multivariate analysis showed that sex was an independent prognostic factor for PFS (HR = 0.746, 95% CI, 0.611–0.910, *P* = 0.004) and OS (HR = 0.755, 95% CI, 0.615–0.926, *P* = 0.007).

**Conclusions:**

This present study indicated that sex was an independent prognostic factor in Chinese patients with ESCC underwent definitive radiotherapy, with better survival outcome for women than men. Efforts should be made to investigate the underlying biological mechanism.

**Electronic supplementary material:**

The online version of this article (10.1186/s13014-019-1278-0) contains supplementary material, which is available to authorized users.

## Introduction

Esophageal cancer (EC) is one of the most common malignant tumors in the world with an estimated 477,900 new cases and the fourth most common cause of cancer-related deaths with 375,000 deaths annually [1]. The incidence of EC significantly vary between men and women, with three to five times more prevalent in men than women [2, 3], which may be due to differences in risk factor exposures and sex hormonal factors [4, 5].

Several retrospective studies have attempted to explore the role of sex on prognosis in patients with ESCC that received definitive chemoradiotheray, but failed to show correlation between sex and prognosis [6, 7]. Therefore, the prognostic value of sex for patients with EC underwent definitive radiotherapy required more investigation.

Many studies have investigated the prognostic value of sex in EC and revealed a sex difference in the prognosis [8–12]. Moreover, gender was found to be an independent prognostic marker in ESCC but not in esophageal adenocarcinoma (EAC) [8, 11, 13], indicating that the effect of sex on prognosis was associated with the histopathological types. However, previous studies that examined the prognostic value of sex were limited by the inadequate adjustment for relevant confounding clinical prognostic factors such as tumor stage, histopathological types, tumor location, treatment modality, intensity of therapy. For patients with EC who had underwent esophagectomy, Kauppila et al. had conducted a population-based nationwide cohort study with long and complete follow-up and adjustment for prognostic factors to clarify whether sex influenced survival [11]. However, for patients with ESCC underwent definitive radiotherapy, studies on the influence of sex on the prognosis are still lacking.

We conducted a retrospective study to evaluate the prognostic value of sex in patients with ESCC underwent definitive radiotherapy using the propensity-scored matching method to adjust for the prognostic factors.

## Patients and methods

### Study design

This was a retrospective study including patients with ESCC who underwent definitive radiotherapy at the Department of Radiation Oncology, Shantou Central Hospital between January 2009 and December 2015, with follow-up until May 31, 2018. Patients that received definitive radiotherapy in this study included three ESCC populations—the patients with inoperable disease, the patients refused surgery for personal willingness, and the patients were not suitable for surgery because of the underlying disease. There weren’t any patients that refused surgery because of their performance status. The intention of treatment was for cure. Inclusion criteria were as followed: 1) pathologically proven ESCC; 2) patients underwent definitive radiotherapy or chemo-radiotherapy. Exclusion criteria were as followed: 1) Adenocarcinoma or other pathological types; 2) Patients with distant metastatic disease; 3) Patients underwent palliative or adjuvant radiotherapy;4) Patients with a poor KPS of 10–50; 5) Patients failed to complete therapy. This study was approved by the Ethics Committee of the Shantou Central Hospital. Patient were staged according to the 6th AJCC TNM classification [14].

### Treatment protocol

All patients underwent definitive radiotherapy delivered by three-dimensional conformal radiation therapy or intensity-modulated radiation therapy technique. The primary gross tumor volume (GTV) and the nodal gross tumor volume (GTVnd) were delineated based on CT, barium esophagogram, endoscopic examination or PET imaging. The clinical target volume (CTV) encompassed tumor clinical target volume (CTVt) and nodal clinical target volume (CTVnd). The CTVt included the GTV plus a radial margin of 0.5 to 1 cm and a proximal and distal margin of 2.5 to 3 cm. The CTVnd included the GTVnd and its expansion margin of 0.5 to 0.8 cm. When developing the radiotherapy plan, the CTV plus a 0.5 to 1 cm expansion margin was defined as the planning target volume (PTV), which the prescribed dose is given to. In this study, patients received a prescribed dose of at least 50.4 Gy. Some patients received chemotherapy consisted of two cycles of platinum-based chemotherapy combined with 5-fluorouracil and a taxane (docetaxel or paclitaxel) concurrently with radiotherapy.

### Follow-up

All patients were examined daily during radiotherapy to monitor the treatment toxicities. The first follow-up was 1 month after completing radiotherapy then every 3–6 months for 5 years. The follow-up evaluation included a physical examination, blood test, barium esophagogram, CT scan of the neck, chest, and abdomen, and PET-CT when available. Information on patients’ age, gender, work-up, treatment, and follow-up was extracted from their medical records.

### Statistical analysis

Progression was defined as the radiographic evidence of relapse, death from any cause. OS was measured from the date of treatment to the date of death from any cause or last follow-up. Comparisons of patient characteristics were carried out with a chi-square test. PFS and OS rates were estimated by the Kaplan-Meier method, and log-rank test was performed to evaluate the survival difference. The Cox regression model was used to perform multivariate analysis to identify prognostic factors. The propensity score-matched analysis (including age, tumor location, T stage, N stage, TNM stage, Treatment modality (definitive radiotherapy or definitive chemoradiotherpy), and radiotherapy dose) was performed using the one-to-one nearest neighbor method. A *P* value < 0.05 indicated a statistical significance. All *P* values were two-sided. The statistical software IBM SPSS v22.0 (SPSS Inc., Chicago, IL, USA) was used for all statistical analysis.

## Results

### Patient characteristics

Between January 2009 and December 2015, a total of 683 patients with ESCC underwent definitive radiotherapy were included in this study, with 497 men and 186 women Additional file 2. All the patients comleted the thereapy. Patient characteristics were summarized in Table 1, and the distribution of age, T stage, N stage, TNM stage and treatment modality between men and women patients were unbalanced. The baseline characteristics were comparative after propensity score-matching (Table 1). Finally, 178 men patients and 178 women patients were included in the propensity score-matched cohort.

**Table 1:** comparison of baseline characteristics between men and women groupts in the original and matched data sets in patients with ESCC

characteristics	Original data set				Matched data set			
	Men(%)	Women(%)	χ2 /t	p	Men(%)	Women(%)	χ2 /t	p
Age (years)	63 (39–89)	68 (40–95)	−3.49	0.001	65.5 (43–89)	67 (40–95)	−1.525	0.128
Location			3.27	0.352			2.54	0.468
Cervical	31 (6.2)	14 (7.5)			17 (9.6)	12 (6.7)		
Upper thoracic	106 (21.3)	45 (24.2)			46 (25.8)	41 (23.0)		
Middle thoracic	291 (58.6)	110 (59.1)			92 (52.8)	108 (60.7)		
Lower thoracic	69 (13.9)	17 (9.1)			21 (11.8)	17 (9.6)		
T stage			12.11	0.007			2.14	0.543
T1	8 (1.6)	6 (3.2)			5 (2.8)	4 (2.2)		
T2	12,024.1)	65 (34.9)			51 (28.7)	62 (34.8)		
T3	135 (27.2)	50 (26.9)			57 (32.0)	47 (26.4)		
T4	234 (47.1)	65 (34.9)			65 (36.5)	65 (36.5)		
N stage			12.51	0.000			0.354	0.552
N0	88 (17.7)	56 (30.9)			46 (25.8)	51 (28.7)		
N1	409 (82.3)	130 (69.1)			132 (74.2)	127 (71.3)		
TNM stage			17.70	0.001			4.35	0.226
I	47 (9.5)	36 (19.4)			29 (16.3)	32 (18.0)		
II	147 (29.6)	64 (34.4)			65 (36.5)	60 (33.7)		
III	290 (58.4)	84 (45.2)			76 (42.7)	84 (47.2)		
IV	13 (2.6)	2 (1.1)			8 (4.5)	2 (1.1)		
Treatment			12.32	0.000			2.23	0.135
RT	193 (38.8)	100 (53.8)			106 (59.6)	92 (51.7)		
CCRT	304 (61.2)	86 (46.2)			72 (40.4)	86 (48.3)		
RT dose (Gy)	64 (50–78)	64 (50–70)	0.44	0.660	64 (50–74)	64 (50–70)	1.552	0.131

### Sex and survival for ESCC

**Fig. 1:**
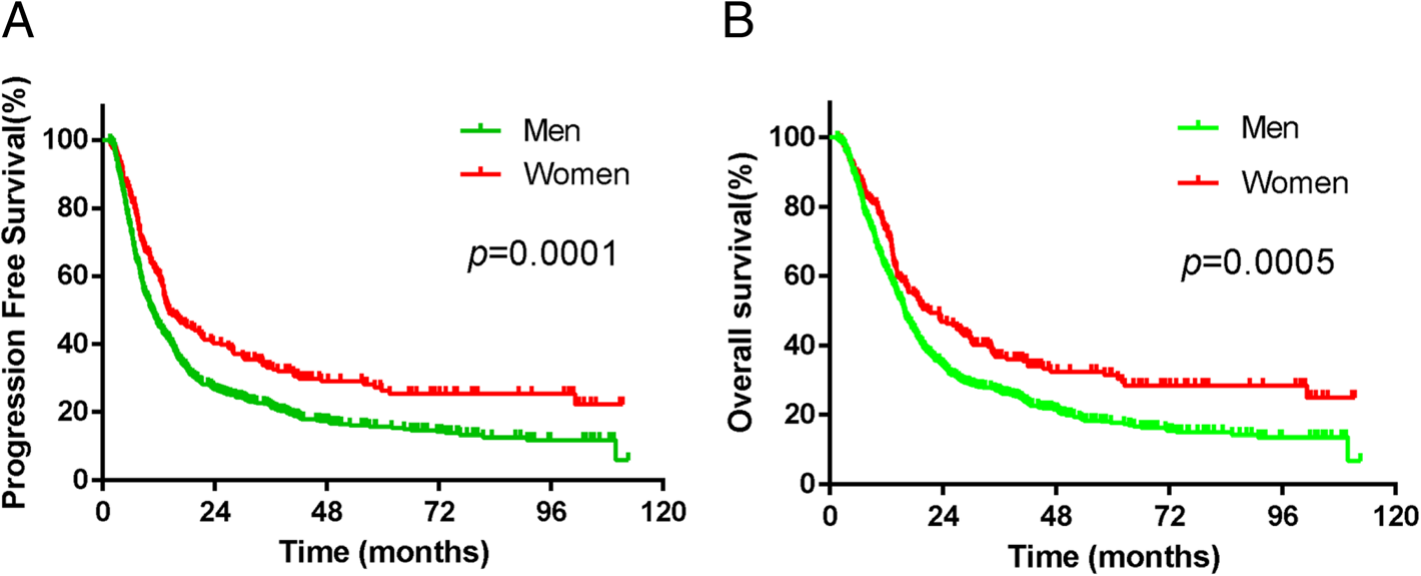
PFS (**a**) and OS (**b**) between men and women patients with ESCC in the whole cohort

Median follow-up was 16.6 months (range, 1.6 to 112.5 months) in the whole cohort, with a median follow-up of 15.9 months (range, 1.6 to 112.5 months) for men and 21.1 months (range, 1.8 to 111.3 months) for women. In the whole cohort, women had a significantly longer median PFS time compared with men patients (14.0 months vs 10.6 months, χ2 = 14.202, *P* = 0.0001, HR = 0.688, 95% CI, 0.567–0.836, Fig. 1a). The 3-and 5-year PFS rates were 33.2 and 26.3% in women and 22.0 and 15.7% in men, respectively. The median OS time was 20.8 months in women, which was significantly longer than 15.9 months in men (χ2 = 12.138, *P* = 0.0005, HR = 0.702, 95% CI, 0.575–0.857, Fig. 1b). The 3-and 5-year OS rates were 36.7 and 31.5% in women and 26.9 and 17.7% in men, respectively.

**Fig. 2:**
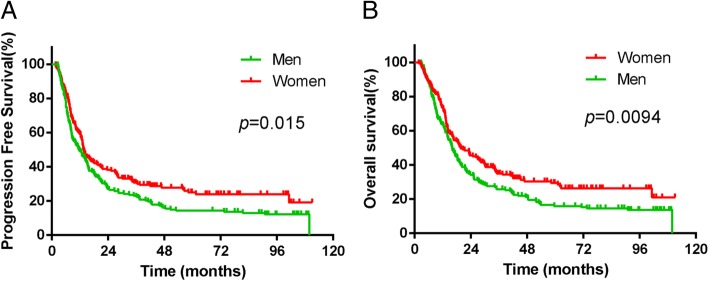
PFS (**a**) and OS (**b**) between men and women patients with ESCC in the matched cohort

These findings were further confirmed in the matched cohortThe median PFS times were 13.5 months (95% CI, 11.436–15.564 months) for women and 11.6 months (95% CI, 8.259–14.941 months) for men (χ2 = 5.910, *P* = 0.015, HR = 0.751, 95% CI, 0.595–0.947, Fig. 2a). The median OS time were 19.6 months (95% CI, 13.571–25.629 months) and 16.1 months (95% CI, 13.776–18.424 months) in women patients and men (χ2 = 6.741, *P* = 0.0094, HR = 0.733, 95% CI, 0.578–0.82, Fig. 2b), respectively.

### Sex and prognostic factors for ESCC

Multivariate analysis showed that sex (HR = 0.746, 95% CI, 0.611–0.910, *P* = 0.004), tumor location (HR = 1.162, 95% CI, 1.031–1.308, *P* = 0.014), T stage (HR = 1.184, 95% CI, 1.029–1.362, *P* = 0.019), N stage (HR = 1.328, 95% CI, 1.013–1.741, *P* = 0.040), TNM stage (HR = 1.236, 95% CI, 1.014–1.506, *P* = 0.036) and treatment modality (HR = 0.741, 95% CI, 0.621–0.884, *P* = 0.001) were independent prognostic factors for PFS (Table 2 and Additional file 1: Table S1), and identified sex (HR = 0.755, 95% CI, 0.615–0.926, *P* = 0.007) as an independent prognostic factor for OS, as well as tumor location (HR = 1.131, 95% CI, 1.003–1.275, *P* = 0.045), T stage (HR = 1.266, 95% CI, 1.095–1.462, *P* = 0.001), RT dose (HR = 0.970, 95% CI, 0.946–0.995, *P* = 0.017), and treatment modality (HR = 0.712, 95% CI, 0.595–0.852, *P* = 0.000).

**Table 2:** Multivariate analysis of clinical factors associated with Progression-Free Survival and Overall Survival among patients with ESCC

Variates	Progression-Free Survival			Overall Survival		
	HR(95%CI)	χ2	p	HR(95%CI)	χ2	p
gender	0.746 (0.611–0.910)	8.375	0.004	0.755 (0.615–0.926)	7.275	0.007
age	0.997 (0.988–1.006)	0.511	0.475	0.997 (0.988–1.006)	0.470	0.493
location	1.162 (1.031–1.308)	6.095	0.014	1.131 (1.003–1.275)	4.023	0.045
T stage	1.184 (1.029–1.362)	5.547	0.019	1.266 (1.095–1.462)	10.22	0.001
N stage	1.328 (1.013–1.741)	4.219	0.040	1.303 (0.993–1.711)	3.640	0.056
TNM stage	1.236 (1.014–1.506)	4.411	0.036	1.181 (0.965–1.445)	2.601	0.107
RT dose	0.977 (0.954–1.001)	3.631	0.057	0.970 (0.946–0.995)	5.681	0.017
Treatment	0.741 (0.621–0.884)	11.061	0.001	0.712 (0.595–0.852)	13.71	0.000

### Sex and recurrence for ESCC

**Fig. 3:**
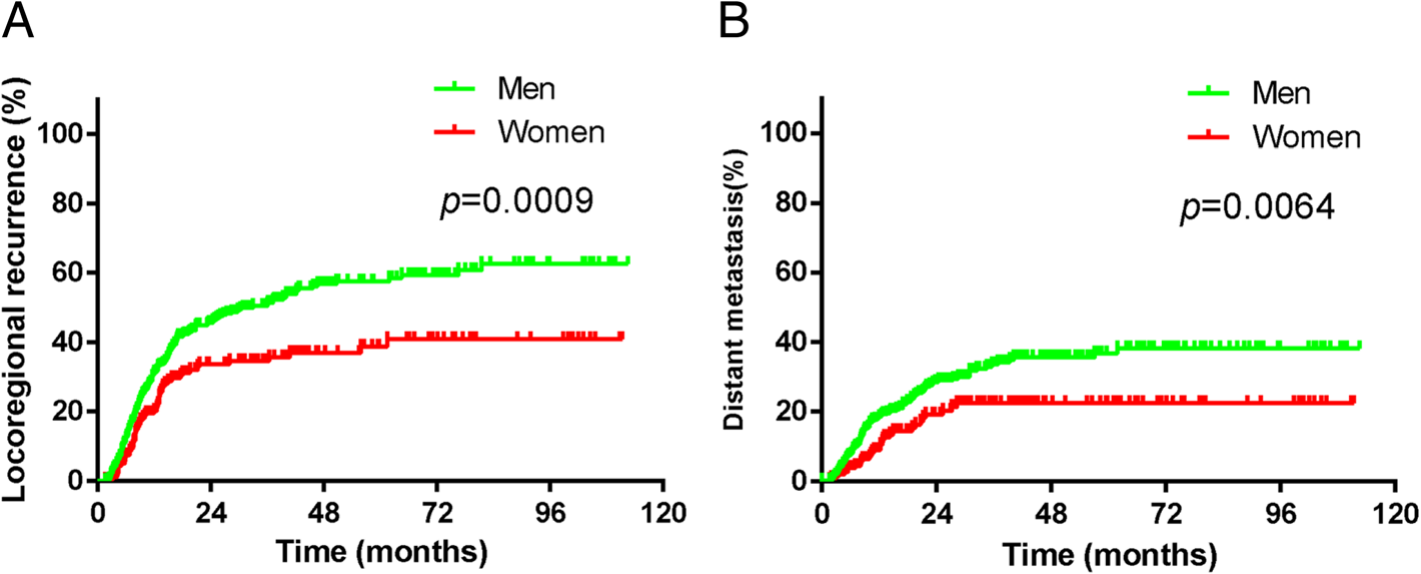
Overall recurrence rate (**a**) and distant metastasis rate (**b**) between men and women patients with ESCC

As shown in Fig. 3a, after a median follow-up of 16.6 months (range, 1.6 to 112.5 months), the logoregional recurrence rate in female patients was significantly lower than that in men (χ2 = 11.11, *P* = 0.0009). A similar finding was observed for distant metastasis event, with fewer patients experiencing distant metastases in female group than in male group (χ2 = 7.446, *P* = 0.0064, Fig. 3b).

## Discussion

ESCC and EAC, the two major histologic subtypes of EC, were different in terms of geographic and demographic characteristics, risk factors, and pathogenesis [15]. ESCC, the predominant subtype of EC, was a great health burden in China due to its poor prognosis [1, 16, 17]. For patients with ESCC who refused surgery or patients with a unresectable disease, definitive radiotherapy or chemo-radiotherapy was the standard treatment [18]. However, most patients treated with definitive chemo-radiotherapy relapsed and finally succumbed to this disease [16, 19, 20]. Therefore, identifying patients who were most likely to benefit from definitive radiotherapy would help to investigate the biological mechanisms affecting the radio-sensitivity of ESCC.

According to statistics, for all cancers combined, women had better survival than men in China [1]. Several studies had evaluated the influence of sex on prognosis in patients with EC [8, 10–12, 21]. Bohanes et al [8] reported the largest register-based study based on the Surveillance, Epidemiology, and End Results (SEER) database and demonstrated that women had better survival in loco-regional ESCC but not in EAC. Another register-based study from Sweden indicated that women had a lower overall all-cause mortality compared with men only in ESCC [11]. Both two studies did not report detailed clinical information about treatment modality. For patients who underwent surgery, women with ESCC were found to have better survival than men in several studies [10, 21]. However, influence of sex on the patients with ESCC who underwent definitive radiotherapy was inadequately studied. In the current study, women with ESCC who underwent definitive radiotherapy had a better PFS and OS than men, and multivariate analysis suggested sex was an independent prognostic factor. Moreover, women had lower overall recurrence and distant metastases. In addition, after balancing clinicopathological prognostic factors including age, tumor location, T stage, N stage, TNM stage, radiotherapy dose, and treatment modality by propensity score matching, better PFS and OS remained in female patients. Taken together, female patients who underwent definitive radiotherapy for ESCC had better prognosis than men.

However, the prognostic significance of sex in ESCC should be interpreted cautiously. Zhang et al. assessed the role of sex on prognosis of ESCC in Chinese and in Caucasian patients in the United States, and found that sex was not an independent prognostic factor in these patients [22]. However, that report didn’t provide specific information about variables and treatment modality which might affect the analysis. In a post hoc analysis that examined the role of sex in Asian patients with ESCC [22], no significant difference in esophageal cancer-specific survival was observed between women and men. Therefore, the role of sex on prognosis of ESCC could not be generalized to different ethnic groups and patients who received different treatments. In the current study, the significance of sex in ESCC was evaluated in the patients who underwent definitive radiotherapy in ChaoShan region in Guangdong Province, China, adding evidence to the effects of sex on the prognosis of ESCC.

Another significant difference observed in the current study was that more male patients experienced regional recurrence and distant metastasis than female patients, indicating a better disease control after radiotherapy in women. However, whether sex affected the radio-sensitivity of ESCC was unclear, a direct influence of sex on radiation sensitivity in ESCC were under-investigated. A retrospective study of patients treated with neoadjuvant chemoradiotherapy (nCRT) followed by surgery may explained the different prognosis between female and male patients treated with definitive radiotherapy [12]. After nCRT, more women (58%) attained a complete or nearly pathologic response compared with men (47%). Moreover, men had an 80% increase in the risk of recurrence. The merits of our study were its sample size and its assessment for the direct influence of sex on prognosis of patients treated with definitive radiotherapy, and identified sex as an indicator of radio-sensitivity to radiotherapy in ESCC.

On the basis of our current data, we hypothesized that sex difference would affect the radio-sensitivity of patients with ESCC exposed to radiotherapy. Following efforts will be made to find out the relation between sex and radio-sensitivity in ESCC. Previous studies have shown that androgen exposure can facilitate the growth of human esophageal squamous cell carcinoma cells and activation of androgen receptors may promote progression of esophageal squamous cell carcinoma [23, 24]. The relation between androgen levels and prognosis of patients with ESCC exposed to radiotherapy will be investigated in our further study. Some experiments in vivo and vitro will be performed to explore the mechanism how androgen affects the radio-sensitivity of esophageal squamous cell carcinoma. Once confirmed, some future clinical research comparing combined chemoradiotherapy and anti-androgenic treatment with chemoradiotherapy alone in ESCC may be considered. Speculatively, intensified chemoradiotherapy would be warranted and anti-androgenic treatment may be added to chemoradiotherapy in male patients with ESCC. But for now, to be honest, evidence was still weak. Consolidation chemotherapy after definitive CRT might be a strategy for male patients.

The current study had several limitations. The primary limitation was that the information about lifestyle factors, socioeconomic status, radiotherapy target, and other prognostic risks was not available. In addition, the retrospective and nonrandomized design limited the strength of the results.

## Conclusion

Our study demonstrated that sex was an independent prognostic factor in Chinese patients with ESCC who underwent definitive radiotherapy, with a better prognosis in female patients than men patients. Efforts should be made to investigate the biological mechanisms for the sex difference in the prognosis of ESCC patients.

## Additional files

Additional file 1: **Table S1.** Multivariate analysis of clinical factors associated with Progression-Free Survival and Overall Survival among patients with ESCC. (DOC 34 kb)

Additional file 2: Flow chart of patients selection. (TIFF 32 kb)

## Abbreviations

CTV: Clinical target volume
CTVnd: nodal clinical target volume
CTVt: tumor clinical target volume
EAC: Esophageal adenocarcinoma
EC: Esophageal cancer
ESCC: Esophageal squamous cell cancer
GTV: Gross tumor volume
GTVnd: nodal gross tumor volume
nCRT: neoadjuvant chemoradiotherapy
OS: Overall survival
PFS: Progression-free survival
PTV: Planning target volume
SEER: Surveillance, Epidemiology, and End Results
