# Supplementary material for: Impact of sex on the prognosis of patients with esophageal squamous cell cancer underwent definitive radiotherapy: a propensity score-matched analysis
Source: Radiat Oncol. 2019 May 2;14:74. doi: 10.1186/s13014-019-1278-0 (PMC6498616; doi:10.1186/s13014-019-1278-0)
Supplement: Supplementary file 1 — Table S1. Multivariate analysis of clinical factors associated with Progression-Free Survival and Overall Survival among patients with ESCC. (DOC 34 kb) [file 13014_2019_1278_MOESM1_ESM.doc]

Additional file 1: Table S1 Multivariate analysis of clinical factors associated with Progression-Free Survival and Overall Survival among patients with ESCC

| Variates | Progression-Free Survival | | | Overall Survival | | |
| --- | --- | --- | --- | --- | --- | --- |
|  | HR（95%CI） | χ2 | p | HR（95%CI） | χ2 | p |
| Location  (reference category lower thoracic) |  |  |  |  |  |  |
| Cervical | 0.738（0.486-1.123） | 2.006 | 0.157 | 0.783（0.512-1.199） | 1.264 | 0.261 |
| Upper thoracic | 0.698（0.517-0.942） | 5.520 | 0.019 | 0.739（0.546-1.000） | 3.841 | 0.050 |
| Middle thoracic | 0.881（0.686-1.129） | 1.003 | 0.317 | 0.889（0.690-1.145） | 0.832 | 0.362 |
| T stage  (reference category T4) |  |  |  |  |  |  |
| T1 | 0.559（0.271-1.152） | 2.487 | 0.115 | 0.475（0.230-0.979） | 4.074 | 0.044 |
| T2 | 0.737（0.550-0.987） | 4.200 | 0.040 | 0.642（0.475-0.868） | 8.289 | 0.004 |
| T3 | 0.719（0.5650.916） | 7.161 | 0.007 | 0.609（0.475-0.781） | 15.250 | 0.000 |
